# Screen‐detected and interval colorectal cancers in England: Associations with lifestyle and other factors in women in a large UK prospective cohort

**DOI:** 10.1002/ijc.32168

**Published:** 2019-02-15

**Authors:** Roger Blanks, Andrea Burón Pust, Rupert Alison, Emily He, Isobel Barnes, Julietta Patnick, Gillian K Reeves, Sarah Floud, Valerie Beral, Jane Green

**Affiliations:** ^1^ Cancer Epidemiology Unit, Nuffield Department of Population Health University of Oxford Headington Oxford United Kingdom; ^2^ Department of Epidemiology and Evaluation Hospital del Mar Barcelona Spain; ^3^ IMIM (Hospital del Mar Medical Research Institute) Barcelona Spain; ^4^ REDISSEC Health Services Research on Chronic Patients Network Madrid Spain; ^5^ Prince of Wales Clinical School UNSW Australia Sydney NSW Australia

**Keywords:** colorectal neoplasms, screening, interval cancer, smoking

## Abstract

Faecal occult blood (FOB) ‐ based screening programmes for colorectal cancer detect about half of all cancers. Little is known about individual health behavioural characteristics which may be associated with screen‐detected and interval cancers. Electronic linkage between the UK National Health Service Bowel Cancer Screening Programme (BCSP) in England, cancer registration and other national health records, and a large on‐going UK cohort, the Million Women Study, provided data on 628,976 women screened using a guaiac‐FOB test (gFOBt) between 2006 and 2012. Relative risks (RRs) and 95% confidence intervals (CIs) were estimated by logistic and Cox regression for associations between individual lifestyle factors and risk of colorectal tumours. Among screened women, 766 were diagnosed with screen‐detected colorectal cancer registered within 2 years after a positive gFOBt result, and 749 with interval colorectal cancers registered within 2 years after a negative gFOBt result. Current smoking was significantly associated with risk of interval cancer (RR 1.64, 95%CI 1.35–1.99) but not with risk of screen‐detected cancer (RR 1.03, 0.84–1.28), and was the only factor of eight examined to show a significant difference in risk between interval and screen‐detected cancers (p for difference, 0.003). Compared to screen‐detected cancers, interval cancers tended to be sited in the proximal colon or rectum, to be of non‐adenocarcinoma morphology, and to be of higher stage.

## Abstract 2

**What's new?**

In the United Kingdom, about half of all colorectal cancers (CRCs) in those who are screened are detected with fecal occult blood (FOB) testing. The remainder of cases are identified in the interval between screening visits. Here, individual health behavioral characteristics were investigated for relationships with CRC detection using UK cohort study data on 628,976 women screened through guaiac‐FOB testing. Risk of interval CRC but not of screen‐detected disease was increased by current smoking. Smoking was the only factor of eight in the study to be associated with differences in risk between interval and screen‐detected CRC.

**Abbreviations**
NHS: National Health Service
FOB(t): faecal occult blood (test)
gFOB(t): guaiac faecal occult blood (test)
RR: relative risk
CI: confidence interval
CRC: colorectal cancer
BCSP: Bowel Cancer Screening Programme
ICD‐10: International Classification of Diseases, 10th revision
ICD‐O 3 (4): International Classification of Diseases for Oncology, 3rd (4th) edition
WHO: World Health Organisation
IARC: International Agency for Research on Cancer
WEO: World Endoscopy Organisation

## Introduction

Colorectal (bowel) cancer is the second most common cancer in women and the third in men worldwide.^1^ In the United Kingdom, bowel cancer is the fourth most common cancer and the second most common cause of cancer death after lung cancer, accounting in 2015/16 for over 41,000 new cases and over 16,000 deaths a year.^2^

Screening with faecal occult blood (FOB) testing aims at detecting and treating early stage tumours and the removal of premalignant lesions, and has been proved in randomised controlled trials to reduce colorectal cancer mortality.^3^ The National Health Service (NHS) Bowel Cancer Screening Programme in England was started in 2006 and invites men and women aged 60–74 to complete a postal guaiac faecal occult blood test (gFOBt) every 2 years, with follow‐up diagnostic testing (usually colonoscopy) for those who are FOB positive.^4^ As in other similar programmes, UK screening sensitivity for cancer is about 50%:^5^ that is, at each screen, about half of all colorectal cancers thought to be present in the screened population give rise to a positive FOB test and are detected. The remainder present symptomatically in the interval between one screen and the next.

Some features of screen‐detected and interval colorectal cancers have been reported from England^6^, ^7^ and from the similar screening programme in Scotland,^5^, ^8^, ^9^ but the screening programmes collect only limited information on individual participant characteristics which may be related to cancer detection. Using data from the NHS Bowel Cancer Screening Programme with electronic linkage to an on‐going large UK prospective cohort, the Million Women Study, we report here tumour characteristics, including stage, grade, location and morphology, and relationship with lifestyle and other personal factors, for colorectal cancers detected at screening and in the interval between screens among 630,000 women screened in England between 2006 and 2012.

## Methods

### The Million Women Study cohort

Between 1996 and 2001, around 1.3 million women aged on average 56 years (SD 5) joined the Million Women Study through National Health Service (NHS) breast screening clinics in England and Scotland, completing a questionnaire about anthropometric, social and demographic factors, and other personal characteristics. The cohort has been resurveyed every 3–5 years since then. The study design and methods are described in detail elsewhere;^10^ study questionnaires, and details of data and access policies, can be viewed online at http://www.millionwomenstudy.org. Women gave consent for follow‐up through their medical records, and the study has NHS ethical approval (East of England, Cambridge South Research Ethics Committee: REC 97/5/001).

All study participants are followed through linkage to NHS central registers for deaths, cancer registrations and hospital admissions. Data for England are provided through NHS Digital. To date, only about 1% of the cohort has been lost to follow‐up, mainly by emigration and cessation of registration with the NHS. Linked cancer registration records available to the study include cancer type and site coded to the 10th Revision of the International Classification of Diseases (ICD‐10), with morphology coded to ICD‐0‐3 or 4. The Million Women Study also has additional information on stage and grade of the majority of registered cancers through linkage to National Cancer Registration and Analysis Service data held by Public Health England.

### The NHS Bowel Cancer Screening Programme

The NHS Bowel Cancer Screening Programme (BCSP) in England sends biennial invitation letters to all men and women aged 60–74 years who are registered with the NHS. About 2 weeks after the invitation letter a gFOBt kit is sent by mail, with instructions on how to use the kit, and return it to the screening programme. Those who test FOB positive are offered further diagnostic tests, which can include colonoscopy (the default test, used for the great majority), flexible‐sigmoidoscopy, and radiological investigations. The screening programme central database holds individual data on screening invitations, acceptance and FOBt results. For those who are FOBt positive, results of further investigations, including cancers detected within the programme, are also recorded, where available (some people do not attend for diagnostic testing within the screening programme after a positive screening test). Screen‐detected cancers diagnosed within the screening programme are also reported to the national cancer registry. Those diagnosed with cancer or advanced precancerous polyps receive appropriate treatment; those diagnosed with intermediate risk pre‐cancerous polyps enter surveillance; those with low‐risk polyps, or no neoplasia, return to routine recall (as do those with negative FOB screening tests).

### Data linkage between Million Women Study and NHS BCSP

Electronic linkage was performed in 2013 by NHS Connecting for Health (now part of NHS Digital), using NHS number and date of birth of Million Women Study participants recruited in England, provided by the study investigators. The data linkage was approved by East of England, Cambridge South Research Ethics Committee and by the NHS Bowel Cancer Screening Programme Research Committee. For these analyses we considered only the first (prevalent) round of bowel cancer screening.

### Outcomes

Colorectal cancers (ICD‐10 C18‐C20) in screened women were identified from national cancer registry data. For the main analyses, screen‐detected colorectal cancer was defined as a first registration for colorectal cancer up to 24 months (or time of next screen, if earlier) after a gFOBt positive screening test (time from the BCSP screening episode start date). Interval cancers were defined according to World Endoscopy Organisation (WEO) proposals^11^ as colorectal cancers registered in the national cancer registry within 24 months (or time of next screen, if earlier) after a negative gFOBt screening test (time from BCSP episode start date). These are thus WEO gFOBt interval cancers within a gFOBt screening programme. For some analyses, results for screen‐detected cancers as recorded in the BCSP were compared to those using cancer registry data.

For analyses by tumour site, cancers were assigned to the following groups: colon, ICD‐10 C18.0–18.9; colon right (proximal), C18.0 caecum to C18.4 transverse colon; colon left (distal), C18.5 splenic flexure to C18.7 sigmoid colon; rectum, C19‐C20. For analyses by morphological type, cancers were divided into 6 groups based on the WHO/IARC classification^12^ (Supporting Information Table SS1): (1) adenocarcinoma, ICD‐O code M8140/3 (and 12 related codes); (2) mucinous adenocarcinoma, ICD‐O M8480/3 (and 3 related codes); (3) signet ring cell carcinoma, ICD‐O code M8490/3; (4) neuroendocrine tumours, including carcinoid, ICD‐O codes M8246/3, M8240/3, M8243/3 and 6 related codes; (5) squamous cell, ICD‐O code M8070/3 (and 2 related codes); and (6) other tumours: all remaining tumours, including other specified carcinoma, specified non‐carcinoma tumours, and cancers of unspecified morphology. Stage of cancer was defined as ‘advanced’ if Dukes stage was C or worse, or TNM stage was 3 or worse.

### Lifestyle and other exposures

For all risk analyses, information on lifestyle, health behaviour and other exposures was obtained from information reported on the Million Women Study recruitment questionnaire. Factors considered were age at screening (<62, 62–63, 64–65, 66–67 and 68+ years, and grouped for some analyses as <65 and 65+ years), socioeconomic status (tertiles of the area‐based Townsend deprivation index^13^), smoking status (never, past, current), body mass index (BMI) (<25, 25+ kg/m^2^, calculated from reported height and weight), parity (nulliparous, parous), past use of oral contraceptives (never, ever), use of hormone therapy (HT) for the menopause (never, ever), strenuous exercise (<1, 1+ times per week) and alcohol intake within drinkers (up to 6, 7+ drinks (~ = units) per week).

### Statistical analysis

Prevalence of screen‐detected colorectal cancer was calculated, assuming that all cancers detected by screening were present at the time of screening (even if not registered until later). Unadjusted incidence rates for FOBt interval cancer, by age, were obtained using follow‐up from the (gFOBt negative) BCSP screening episode start date for 2 years or until the first of date of death, emigration, next bowel cancer screening programme invitation, or date of diagnosis of any other cancer, if sooner. Screening sensitivity was estimated as a proportion, with the number of screen‐detected cancers as the numerator, and the number of screen‐detected plus interval cancers as the denominator, i.e. assuming that the vast majority of interval cancers occurring within 2 years of a negative FOBt test were missed by the screening test as a cancer, or at least as an advanced pre‐invasive lesion.^8^ To study factors associated with screen detected cancer, we calculated adjusted risk ratios (henceforth referred to as relative risks, RRs) and 95% confidence intervals (CIs) using logistic regression. For interval cancers, which may occur throughout the 2 year follow‐up period, Cox proportional hazards regression was used to calculate multivariate adjusted hazard ratios (also referred to here as relative risks) and their 95% CIs in relation to lifestyle and other exposure factors of interest. For all risk factor analyses, women with missing values of any of the adjustment variables were assigned to a separate category for that variable. All analyses were adjusted for age and region, and as appropriate for all other variables in the model. Risk factor sensitivity analyses were conducted, (a) excluding women with a record of any cancer (other than non‐melanoma skin cancer, C44) prior to invitation to screening; and (b) defining screen‐detected cancers as those recorded as such within the BCSP. Competing risks methods were used to compare relative risks between screen‐detected and interval cancers. All analyses used Stata version 14.^14^

## Results

A total of 899,166 Million Women Study participants in the linked dataset received at least one invitation from the NHS BCSP in England for screening. These women had their first invitation for routine bowel cancer screening between December 2006 and March 2012 (mean year 2009, SD 1.1) at a mean age of 65.3 (SD 3.6) years. Of the 899,166 women invited 628,976 (70.0%) participated in the first screening round, completing a screening test with a definitive FOB test result (9,133 [1.5%] of which were positive). These women form the study population for these analyses. Of those testing FOB positive, 7,911 women (87%) completed diagnostic investigations within the BCSP.

Among all screened women, 766 had screen‐detected colorectal cancer identified in the cancer registry within 24 months of a positive gFOBt result (93% within 6 months, and 98% within 12 months), and 749 had interval colorectal cancers registered within 2 years of a negative gFOBt result (12% within 6 months, 36% within 12 months). The overall prevalence of screen detected cancers was 1.22 per 1,000 women screened, with an increase in prevalence with age. FOBt interval cancers occurred at an overall rate of 0.60 per 1,000 person‐years, with the rate similarly increasing with increasing age at screening, and higher in the second year after screening (0.76 per 1,000 person‐years) than in the first (0.44 per 1,000 person‐years) (Supporting Information Table S2). The estimated sensitivity of screening was 51% (95% CI, 48%–53%).

Screen‐detected cancers were more likely to be found in the left colon and interval cancers in the right colon; screen detected cancers were also more likely to be of less advanced stage (Table 1). Interval cancers were somewhat more likely to be of morphologies other than adenocarcinoma; in particular, interval cancers accounted for the great majority of neuroendocrine tumours. Estimated screening sensitivity varied little by age but was notably higher (65%) for cancers of the left colon than of the right colon (40%) or rectum (49%). The great majority of colorectal cancers were classed as adenocarcinomas, and estimates of sensitivity by morphological type are limited by small numbers of rarer types, but the results suggest that sensitivity may be higher for adenocarcinoma than for tumours of other morphologies.

**Table 1:** Characteristics of screen‐detected and interval cancers in women in the NHS Bowel Cancer Screening Programme in England.

	Screen‐detected cancers, n (%)	Interval cancers n (%)	Estimated sensitivity of screening, % (95% CI)
All	766 (100%)	749 (100%)	51 (48–53)
			
*Age at screening, years*			
<65	271 (35%)	280 (37%)	49 (45–53)
65+	495 (65%)	469 (63%)	51 (48–55)
*Tumour location*			
Right Colon	223 (29%)	336 (45%)	40 (36–44)
Left colon	331 (43%)	178 (24%)	65 (61–69)
Rectum	211 (28%)	224 (30%)	49 (44–53)
missing	<5	11 (1%)	
*Grade*			
1 or 2	519 (68%)	422 (56%)	55 (52–58)
3 or 4	86 (11%)	98 (13%)	47 (39–54)
missing	161 (21%)	229 (31%)	
*Stage* 1			
Not advanced	326 (43%)	231 (31%)	59 (54–63)
Advanced	218 (28%)	304 (41%)	42 (37–46)
missing	222 (29%)	214 (29%)	
*Morphology*			
Adenocarcinoma	612 (80%)	531 (71%)	54 (51–56)
Mucinous	33 (4%)	42 (6%)	44 (33–56)
Signet ring	<5	<5	
Neuroendocrine	<5	18 (2%)	
Squamous	<5	7(1%)	
Other/missing	118 (15%)	147 (20%)	

1 Stage defined as ‘advanced’ if Dukes stage C or worse, or TNM stage 3 or worse.

Table 2 shows adjusted relative risks and 95% confidence intervals for screen detected and interval cancers in relation to eight selected factors. For both screen‐detected and interval colorectal cancers, risk was modestly increased with higher body mass index and higher alcohol consumption, and modestly reduced with higher levels of exercise, in parous compared to nulliparous women, and in women using hormone therapy for menopause. Socioeconomic status and use of oral contraceptives were not associated with either group of cancers. Current smoking was significantly associated with risk of interval cancer (RR 1.64, 95%CI 1.35–1.99) but not with risk of screen‐detected cancer (RR 1.03, 0.84–1.28), and was the only factor examined to show a significant difference in risk between interval and screen‐detected cancers (p for difference, 0.003). Risk factor associations were very similar in analyses in which women with prior cancer were excluded, and when the BCSP record, rather than the cancer registry, was used to identify screen‐detected cancers (Supporting Information Tables S3 and S4).

**Table 2:** Risk of screen‐detected and interval colorectal cancers in relation to health behaviour and other factors in women in the NHS Bowel Cancer Screening Programme in England.

	Screen‐detected cancer		Interval cancer		Interval *vs* Screen‐Detected
	n cases 766	Relative Risk (95%CI)	n cases 749	Relative Risk (95%CI)	*P for difference*
*Socioeconomic status (tertiles)*					
Least deprived	411	1.00	373	1.00	
Most deprived	352	0.95 (0.82–1.09)	372	1.09 (0.94–1.27)	*P = 0.22*
*Body mass index, kg/m* ^*2*^					
<25	311	1.00	332	1.00	
25+	412	1.24 (1.07–1.44)	382	1.11 (0.96–1.29)	*P = 0.36*
*Strenuous exercise*					
<once per week	465	1.00	439	1.00	
Once + per week	282	0.85 (0.73–0.99)	292	0.95 (0.82–1.10)	*P = 0.32*
*Smoking status*					
Never	375	1.00	331	1.00	
Past	242	1.16 (0.99–1.37)	223	1.20 (1.01–1.42)	
Current	115	1.03 (0.84–1.28)	163	1.64 (1.35–1.99)	*P = 0.003*
*Alcohol (in drinkers)*					
<7drinks/pw	389	1.00	374	1.00	
7+ drinks/pw	210	1.22 (1.03–1.44)	209	1.19 (1.00–1.42)	*P = 0.92*
*Full term pregnancy*					
Never	84	1.00	94	1.00	
Ever	679	0.91 (0.72–1.14)	653	0.76 (0.61–0.95)	*P = 0.41*
*Hormone Therapy use*					
Never	377	1.00	338	1.00	
Ever	382	0.80 (0.70–0.93)	403	0.93 (0.80–1.07)	*P = 0.21*
*Oral contraceptive use*					
Never	285	1.00	255	1.00	
Ever	473	1.04 (0.89–1.21)	488	1.16 (0.99–1.36)	*P = 0.52*

Of the 766 cancers identified as screen‐detected using cancer registry data, 706 were also recorded in BCSP as screen‐detected cancers (i.e., cancers diagnosed as a direct result of diagnostic testing within the screening programme). Of these, 83% were recorded within 6 months and 97% within 12 months of the screening episode start date. A further 37 cancers appeared in BCSP records as screen‐detected, but not in the cancer registry; and 60 cancers in women with FOBt positive tests were recorded in the registry but not in screening programme records. The majority of these (49/60) were in women who did not have diagnostic follow‐up within the screening programme after a positive gFOBt, and the cancers tended to be registered later than the other cancers in FOBt positive women (Table 3). Screening sensitivity estimated using BCSP records only to identify screen‐detected cancers was 50% (47%–52%); and using all available records, 52% (49%‐ 54%) (Supporting Information Table S5).

**Table 3:** Colorectal cancers identified in cancer registry and in Bowel Cancer screening Programme (BCSP) records, in the 2 years after start of screening episode, by result of screening FOB test.

Time from screening episode start	0–6	6–12	12–24^1^	TOTAL
	months	months	months	
*Screening test category*				
*CRC in cancer registry*				
all FOBT –ve	92 (12%)	182 (24%)	475 (63%)	749
all FOBT +ve	711 (93%)	40 (5%)	15 (2%)	766
FOBT +ve, attended screening diagnostic test	683 (95%)	26 (4%)	8 (1%)	717
FOBT +ve, did not attend screening diagnostic test	28 (57%)	14 (29%)	7 (14%)	49
				
*CRC in screening programme record (BCSP)*				
FOBT +ve, attended screening diagnostic test	609 (83%)	113 (15%)	20 (3%)	743^2^

1 Or to next screen, if <24 months.

2 Includes one cancer recorded in BCSP after 24 m but before next screen.

## Discussion

Linkage of the large Million Women Study cohort to the NHS Bowel Cancer Screening Programme in England, with follow‐up through national cancer and death registries, has allowed us to compare individual lifestyle factors associated with risk of screen‐detected and interval cancers in a population‐based gFOBt screening programme. Among screened women, current (but not past) smokers were at significantly increased risk of interval, but not of screen‐detected, colorectal cancer. No significant differences were seen between risks of screen‐detected and interval cancers for socioeconomic status, body mass index, alcohol consumption, physical activity, parity, use of hormone therapy for menopause or past use of oral contraceptives.

UK and other studies of population‐based bowel cancer screening programmes^5^, ^6^, ^7^, ^8^, ^9^, ^15^, ^16^, ^17^ have consistently reported that interval cancers following negative faecal occult blood screening tests (both gFOBt and faecal immunochemical tests, FIT) tend to be found more often in women, in the right (proximal) colon and rectum, and to be of higher stage than cancers detected by screening; our results for site and stage are consistent with their findings (as is the overall sensitivity of screening). Our results also suggest that cancers of rarer morphologies (mucinous, signet ring cell, neuroendocrine and squamous) are more likely to be detected as interval cancers.

The UK cancer screening programmes do not routinely collect individual data on health and lifestyle factors such as smoking and body size, and relevant previous reports from the English^6^, ^7^ and Scottish^5^, ^8^, ^9^ FOB‐based bowel cancer screening programmes have included individual data only on socioeconomic status, age and sex. These studies found, as we did, little or no evidence for differences by socioeconomic status between screen‐detected and interval cancers. Our main results are based on cancers registered in the national cancer registration database (and are thus directly comparable with cancers reported from Scotland, where the screening programme does not itself undertake diagnostic tests). We found that English BCSP records of cancers diagnosed among women who attended screening programme diagnostic testing were largely consistent with registered cancers, and the results of our risk factor analyses were similar when using BCSP records to identify screen‐detected cancers (as in previous reports for the English screening programme).

Cancers which present in the interval between screening tests probably represent largely cancers which (as cancer or precursor) were present, but were missed by the screening or subsequent diagnostic test. For FOB programmes, this may include tumours which either do not bleed sufficiently for detection, or whose location (more proximal, or rectal) or gross morphology mean that it is less likely either that faecal haemoglobin is present in a detectable form, or that the lesion is detected at diagnostic colonoscopy.^5^ Some may also be fast‐growing, more aggressive lesions which grow to symptomatic size within the 2 year screening interval, although interval cancers as a whole, when compared to cancers detected in an unscreened population, are not reported to be especially large or of higher stage; there are conflicting reports on whether they are or are not associated with worse biological prognostic characteristics or short term survival.^8^, ^9^, ^18^, ^19^, ^20^ Our finding that smoking is related to risk of gFOBt interval, but not of screen‐detected, cancers is consistent with the emerging picture of molecular/morphological type‐specific tumours which appear to behave differently both in terms of aetiology, and of screening. We and others have previously found stronger associations for smoking with risk of colorectal cancers other than those coded as adenocarcinoma.^21^ Evidence from molecular epidemiological studies also suggests that smoking may be a risk factor specifically, or largely, for cancers arising from serrated polyps through pathways associated with microsatellite instability and DNA methylation rather than through the ‘conventional’ adenoma‐adenocarcinoma (chromosome instability) pathway.^22^ Serrated pathway cancers, which tend to be sited proximally, may be less likely to bleed and perhaps more likely to be fast‐growing, and faecal occult blood screening has been shown to perform relatively poorly for their detection.^23^, ^24^

This study's strengths include its large size, virtually complete follow‐up through national registers for deaths and cancers, and unique record linkage combining detailed prospectively‐collected information on individual health behavioural factors with screening programme data. Information was available for tumour site, morphology and for many tumours, also for stage and grade. We were able to allow for major potential confounding factors in the analyses. However, even in this very large study the numbers of cancers arising within a period of 2 years was relatively small, and not sufficient for detailed comparisons of risk factors by tumour morphology or site. We had no information on molecular characteristics of tumours. The Million Women Study recruited some 1 in 4 of all women of eligible age in the UK, and results from the study should be generalizable to women in the UK and other similar populations, but, clearly, not necessarily to men or to cancers in populations with very different health care systems. Our results describe risk associations for screen‐detected and FOB‐interval cancers in an FOB‐based programme, and may not apply to cancers arising in the interval after a negative colonoscopy, whether screening or diagnostic (WEO colonoscopy (CS) interval cancers,^11^ also known as ‘post‐colonoscopy colorectal cancers’ or ‘missed cancers’). Such cancers do however appear to share with FOB‐interval cancers some features such as a tendency to arise in the proximal colon, and to show molecular and morphological characteristics of the ‘serrated pathway’^25^, so it may be that they also share common behavioural risk factors with FOB‐interval tumours. There were too few cancers arising in the interval after a negative colonoscopy in our study to allow us to examine risk factor associations in this group.

The rather high rates of interval cancer observed in FOB‐based screening programmes for colorectal cancer are of concern: as well as reflecting low programme sensitivity, false re‐assurance after a negative FOB screening test may lead to delayed symptomatic presentation in the interval between screens.^26^ Population uptake of FOB‐based bowel cancer screening tends to be low (50–60% for UK gFOBt programmes; somewhat higher for FIT‐based). Efforts to improve both uptake and test sensitivity, to maximise programme benefit, are a focus of much current work and underlie the increasing use of FIT‐based tests in the UK. Use of quantitative immunochemical (FIT) rather than guaiac FOB tests allows for higher screening test sensitivity (and correspondingly lower rates of interval cancer), but high sensitivity may in practice be constrained both by health service colonoscopy capacity, and by higher rates of false positive tests and potentially of over‐diagnosis.^5^, ^27^ Individual risk stratification for screening may be an option, with the established lower sensitivity of FOB testing in women and in older people offering an opportunity, for example, to consider FIT test cut‐off levels tailored to maximise test sensitivity for population sub‐groups. Information on individual level risk factors such as smoking has the potential to contribute to such programme improvements.

## Supporting information


**Table S1 ICD‐O codes used for colorectal cancer morphology grouping**
**Table S2 Colorectal cancers recorded in the cancer registry within 24 months of screening**
**Table S3:** Sensitivity analysis: Risk of screen‐detected and interval colorectal cancers in relation to health behaviour and other factors in women in the NHS Bowel Cancer Screening Programme in England, in women with no prior cancer
**Table S4:** Sensitivity analysis: Risk of screen‐detected and interval colorectal cancers in relation to health behaviour and other factors in women in the NHS Bowel Cancer Screening Programme in England, using NHS Bowel Cancer Screening programme (BCSP) records for screen‐detected cancer (n = 743) and cancer registry records for interval cancer (n = 749).
**Table S5: Sensitivity of screening for colorectal cancer in England, estimated using NHS Bowel Cancer Screening Programme and national cancer registration records**
