# Supplementary material for: Screen‐detected and interval colorectal cancers in England: Associations with lifestyle and other factors in women in a large UK prospective cohort
Source: Int J Cancer. 2019 Feb 15;145(3):728–34. doi: 10.1002/ijc.32168 (PMC6563087; doi:10.1002/ijc.32168)
Supplement: Supplementary file 1 — Table S1 ICD‐O codes used for colorectal cancer morphology grouping Table S2 Colorectal cancers recorded in the cancer registry within 24 months of screening Table S3: Sensitivity analysis: Risk of screen‐detected and interval colorectal cancers in relation to health behaviour and other factors in women in the NHS Bowel Cancer Screening Programme in England, in women with no prior cancer Table S4: Sensitivity analysis: Risk of screen‐detected and interval colorectal cancers in relation to health behaviour and other factors in women in the NHS Bowel Cancer Screening Programme in England, using NHS Bowel Cancer Screening programme (BCSP) records for screen‐detected cancer (n = 743) and cancer registry records for interval cancer (n = 749). Table S5: Sensitivity of screening for colorectal cancer in England, estimated using NHS Bowel Cancer Screening Programme and national cancer registration records [file IJC-145-728-s001.docx]

Supplementary **Table 1: ICD-O codes used for colorectal cancer morphology grouping**

| **Group** | **ICD-O code** | **Screen-detected** | **Interval** |
| --- | --- | --- | --- |
| **Adenocarcinoma**  Adenocarcinoma NOS  Other Adenocarcinoma:  Adenocarcinoma, intestinal type  Adenocarcinoma in adenomatous polyp  Tubular adenocarcinoma  Adenocarcinoma in villous adenoma  Villous adenocarcinoma  Adenocarcinoma in tubulovillous adenoma  Papillary adenocarcinoma NOS  Adenocarcinoma in multiple adenomatous polyps  Scirrhous adenocarcinoma  Solid adenocarcinoma NOS  Adenocarcinoma, mixed subtypes  Superficial spreading adenocarcinoma | M8140/3  M8144/3  M8210/3  M8211/3  M8261/3  M8262/3  M8263/3  M8260/3  M8221/3  M8141/3  M8230/3  M8255/3  M8143/3 | **Total: 612**  485  127 | **Total: 531**  477  54 |
| **Mucinous adenocarcinoma**  Mucinous adenocarcinoma  Mucin-producing adenocarcinoma  Mucinous cystadenocarcinoma | M8480/3  M8481/3  M8470/3  M8472/3 | **Total: 33** | **Total: 42** |
| **Signet Ring cell carcinoma** | M8490/3 | **Total: <5** | **Total: <5** |
| **Neuroendocrine tumours**  Neuroendocrine NOS  Carcinoid NOS  Goblet cell carcinoid:  Carcinoid, argentaffin  Composite carcinoid  Adenocarcinoid  Large cell carcinoma, NOS  Small cell carcinoma, NOS  Large cell neuroendocrine carcinoma | M8246/3  M8240/3  M8243/3  M8241/3  M8244/3  M8245/3  M8012/3  M8041/3  M8013/3 | **Total:<5** | **Total: 18** |
| **Squamous**  Squamous carcinoma  Squamous keratinising  Squamous Large | M8070/3  M8071/3  M8072/3 | **Total: <5** | **Total: 7** |
| **Other tumours** |  | **Total: 118** | **Total: 147** |
| **TOTAL** |  | **766** | **749** |

Supplementary **Table 2**

**Colorectal cancers recorded in the cancer registry within 24 months of screening**

| **Age, years** | **Number of women Screened** | **Colorectal cancers following FOBt +ve**  **(screen-detected)**  **n ( /1000)** | **Colorectal Cancers following FOBt –ve**  **(interval cancers)** | | | | | | | | |
| --- | --- | --- | --- | --- | --- | --- | --- | --- | --- | --- | --- |
|  |  |  | **Cases 0-12 mths** | **Person-years** | **Rate**  **/ 1000 pyr** | **Cases 12-24 mths** | **Person-years** | **Rate/ 1000 pyr** | **All cases** | **Person-years** | **Rate**  **/ 1000 pyr** |
| **<62** | 122,865 | 84 (0.68) | 34 | 122,845 | 0.28 | 56 | 122,384 | 0.46 | 90 | 245,228 | 0.37 |
| **62-63** | 129,685 | 117 (0.90) | 46 | 129,650 | 0.35 | 68 | 129,116 | 0.53 | 114 | 258,766 | 0.44 |
| **64-65** | 111,341 | 118 (1.06) | 54 | 111,303 | 0.49 | 85 | 110,823 | 0.77 | 139 | 222,126 | 0.63 |
| **66-67** | 101,257 | 144 (1.42) | 40 | 101,223 | 0.40 | 92 | 100,764 | 0.91 | 132 | 201,987 | 0.65 |
| **68+** | 163,828 | 303 (1.85) | 100 | 163,731 | 0.61 | 174 | 163,357 | 1.07 | 274 | 327,087 | 0.84 |
|  |  |  |  |  |  |  |  |  |  |  |  |
| **Total** | **628,976** | **766 (1.22)** | **274** | **628,751** | **0.44** | **475** | **626,443** | **0.76** | **749** | **1,255,194** | **0.60** |

FOBt = faecal occult blood test; pyr = person years

Supplementary **Table 3:** Sensitivity analysis: Risk of screen-detected and interval colorectal cancers in relation to health behaviour and other factors in women in the NHS Bowel Cancer Screening Programme in England, in women with no prior cancer

|  | **Screen-detected cancers** | | **Interval cancers** | | **Interval vs Screen-Detected** |
| --- | --- | --- | --- | --- | --- |
|  | n cases  723 | Relative Risk (95%CI) | n cases  699 | Relative Risk (HR) (95%CI) | p-value: case-case analysis |
| **Socioeconomic level (tertiles)** |  |  |  |  |  |
| Least deprived | 386 | 1.00 | 348 | 1.00 |  |
| Most deprived | 335 | 0.96 (0.82,1.11) | 347 | 1.09 (0.94,1.27) | *P=0.24* |
| **Body mass index (kg/m^2^)** |  |  |  |  |  |
| <25 | 299 | 1.00 | 307 | 1.00 |  |
| 25+ | 384 | 1.20 (1.03,1.40) | 359 | 1.14 (0.97,1.33) | *P=0.70* |
| **Strenuous exercise** |  |  |  |  |  |
| 0-1 hrs/wk | 437 | 1.00 | 413 | 1.00 |  |
| 2+ hrs/wk | 268 | 0.86 (0.74,1.01) | 271 | 0.93 (0.80,1.09) | *P=0.54* |
| **Smoking status** |  |  |  |  |  |
| Never | 349 | 1.00 | 311 | 1.00 |  |
| Past | 232 | 1.20 (1.01,1.42) | 207 | 1.18 (0.99,1.41) |  |
| Current | 111 | 1.07 (0.86,1.33) | 152 | 1.64 (1.34,2.00) | *P=0.009* |
| **Alcohol** |  |  |  |  |  |
| <7units/pw | 368 | 1.00 | 348 | 1.00 |  |
| 7+ units/pw | 196 | 1.19 (1.00,1.42) | 197 | 1.22 (1.02,1.45) | *P=0.85* |
| **Full term pregnancy** |  |  |  |  |  |
| Never | 78 | 1.00 | 81 | 1.00 |  |
| Ever | 643 | 0.91 (0.72,1.15) | 616 | 0.82 (0.65,1.03) | *P=0.74* |
| **HT use** |  |  |  |  |  |
| Never | 348 | 1.00 | 323 | 1.00 |  |
| Ever | 369 | 0.84 (0.73,0.98) | 370 | 0.89 (0.76,1.03) | *P=0.69* |
| **Oral contraceptive use** |  |  |  |  |  |
| Never | 267 | 1.00 | 228 | 1.00 |  |
| Ever | 449 | 1.04 (0.89,1.22) | 465 | 1.23 (1.04,1.46) | *P=0.26* |

Supplementary **Table 4:** Sensitivity analysis: Risk of screen-detected and interval colorectal cancers in relation to health behaviour and other factors in women in the NHS Bowel Cancer Screening Programme in England, using NHS Bowel Cancer Screening programme (BCSP) records for screen-detected cancer (n=743) and cancer registry records for interval cancer (n=749).

|  | **Screen-detected colorectal cancer**  **recorded in BCSP following FOB positive screening test and BCSP diagnostic test** | | **Interval colorectal cancer**  **recorded in cancer registry following FOB negative screening test** | | **Interval vs Screen-Detected** |
| --- | --- | --- | --- | --- | --- |
|  | n cases  743 | Relative Risk (95%CI) | n cases  749 | Relative Risk (95%CI) | *p-value: case-case analysis* |
| **Socioeconomic level (tertiles)** |  |  |  |  |  |
| Least deprived | 400 | 1.00 | 373 | 1.00 |  |
| Most deprived | 340 | 0.95 (0.82-1.10) | 372 | 1.09 (0.94-1.27) | *P=0.22* |
| **Body mass index (kg/m^2^)** |  |  |  |  |  |
| <25 | 312 | 1.00 | 332 | 1.00 |  |
| 25+ | 391 | 1.18 (1.01-1.37) | 382 | 1.11 (0.96-1.29) | *P=0.64* |
| **Strenuous exercise** |  |  |  |  |  |
| <1 per week | 448 | 1.00 | 439 | 1.00 |  |
| 1+ per week | 276 | 0.86 (0.74-1.00) | 292 | 0.95 (0.82-1.10) | *P=0.38* |
| **Smoking status** |  |  |  |  |  |
| Never | 367 | 1.00 | 331 | 1.00 |  |
| Past | 231 | 1.13 (0.96-1.34) | 223 | 1.20 (1.01-1.42) |  |
| Current | 110 | 1.01 (0.81-1.26) | 163 | 1.64 (1.35-1.99) | *P=0.002* |
| **Alcohol** |  |  |  |  |  |
| <7units/pw | 377 | 1.00 | 374 | 1.00 |  |
| 7+ units/pw | 209 | 1.25 (1.05-1.49) | 209 | 1.19 (1.00-1.42) | *P=0.69* |
| **Full term pregnancy** |  |  |  |  |  |
| Never | 86 | 1.00 | 94 | 1.00 |  |
| Ever | 654 | 0.86 (0.68-1.08) | 653 | 0.76 (0.61-0.95) | *P=0.62* |
| **HT use** |  |  |  |  |  |
| Never | 367 | 1.00 | 338 | 1.00 |  |
| Ever | 369 | 0.80 (0.69-0.92) | 403 | 0.93 (0.80-1.07) | *P=0.20* |
| **Oral contraceptive use** |  |  |  |  |  |
| Never | 280 | 1.00 | 255 | 1.00 |  |
| Ever | 455 | 1.02 (0.87-1.19) | 488 | 1.16 (0.99-1.36) | *P=0.41* |

Supplementary **table 5: Sensitivity of screening for colorectal cancer in England, estimated using NHS Bowel Cancer Screening Programme and national cancer registration records**

| Index Screening Test Result | colorectal cancer identified in cancer registry for all women | | colorectal cancer identified in BCSP for FOBt positive women and in cancer registry for FOBt negative women | | colorectal cancer identified in registry and/or BCSP for FOBt positive women and in cancer registry for FOBt negative women | |  |
| --- | --- | --- | --- | --- | --- | --- | --- |
|  | Cancer | No cancer | Cancer | No cancer | Cancer | No cancer | **total** |
| FOBt positive | 766 | 8,367 | 743 | 8,390 | 803 | 8,330 | 9,133 |
| FOBt negative | 749 | 619,094 | 749 | 619,094 | 749 | 619,094 | 619,843 |
|  |  |  |  |  |  |  |  |
| Total | 1,515 | 627,461 | 1,492 | 627,484 | 1,552 | 627,424 | 628,976 |
|  |  |  |  |  |  |  |  |
| Estimated sensitivity of index screen | 51% (95% CI 48-53%) | | 50% (95% CI 47-52%) | | 52% (95% CI 49-54%) | |  |
